# Plankton Communities Behave Chaotically Under Seasonal or Stochastic Temperature Forcings

**DOI:** 10.1002/ece3.71930

**Published:** 2025-08-16

**Authors:** Guido Occhipinti, Cosimo Solidoro, Roberto Grimaudo, Davide Valenti, Paolo Lazzari

**Affiliations:** ^1^ National Institute of Oceanography and Applied Geophysics – OGS Trieste Italy; ^2^ Dipartimento di Matematica e Geoscienze Università Degli Studi di Trieste Trieste Italy; ^3^ National Biodiversity Future Center (NBFC) Palermo Italy; ^4^ Department of Physics and Astronomy “E. Majorana” University of Catania Catania Italy; ^5^ Dipartimento di Fisica e Chimica “Emilio Segrè” Università Degli Studi di Palermo Palermo Italy

**Keywords:** chaos, plankton, seasonal, stability, stochastic

## Abstract

Chaos is observed in natural systems, especially in ecosystems with populations characterized by a short reproductive time scale, such as plankton. We examined if external forcings can be the origin of chaos in a state‐of‐the‐art marine biogeochemical model. Both a seasonal temperature cycle and noise added to this cycle were studied as external forcings, and their interaction with the dynamics resulting from biological interactions, such as competition. Our findings indicate that deterministic periodic forcing can induce chaos in the ecosystem only when biological interactions have already established periodic oscillations within the system. We conclude that random temperature fluctuations can be a driver of chaos in plankton populations, due also to the stochastic nature of the marine environment. The stochastic mechanism for the occurrence of chaos could find wide application in natural systems as it requires no preconditions.

## Graphical abstract

Periodic forcing can drive a system into chaos when the autonomous system exhibits periodic behavior. In contrast, stochastic forcing has the potential to induce chaos regardless of the underlying dynamics of the autonomous system.

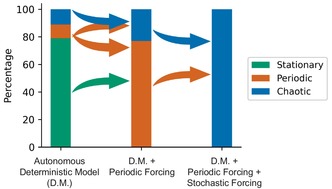

## 1 Introduction

Chaos is observed in natural populations and is especially common in plankton and insects (Rogers et al. 2022). It is defined as bounded, deterministic, aperiodic dynamics that depend sensitively on initial conditions, and it was introduced in the ecological context to explain fluctuations observed in population densities by means of simple low‐dimensional nonlinear models (Dennis et al. 2001). Indeed, aperiodic fluctuations are found in various animal populations (Krebs and Myers 1974; Watson et al. 1984; Berryman 1988; Hudson et al. 1992; Moss et al. 1996) and their understanding intrigued theoretical ecologists since the time of Lotka and Volterra (Kendall et al. 1999). On the other hand, chaos affects the long‐term predictability of ecosystem states (Hastings et al. 1993; Rinaldi and Solidoro 1998) and it has been pointed to as a fundamental factor in sustaining diversity (Huisman and Weissing 1999; Chesson 2020). Therefore, a better understanding of the origin of chaos might be relevant for both conceptual and practical (managerial) reasons.

Within plankton communities, chaotic behavior has been detected in chemostats (Becks et al. 2005), mesocosms (Benincà et al. 2008; Telesh et al. 2019), and even in the field (Benincà et al. 2015; Medvinsky et al. 2015; Rogers et al. 2023). Recent analysis of plankton time series from lakes and marine sites has revealed that chaos in plankton dynamics is widespread (Rogers et al. 2023), but there are still discussions about whether such dynamics arise from biological interactions within the system (endogenous), are related to changes in the environment (exogenous), or are a combination of both. Marine systems from the surface up to a few hundred meters deep undergo the influence of periodic forcings and noise fluctuations, and it is difficult to isolate the endogenous component from the external forcings. Contrasting considerations have been proposed to explain whether an observed process can be classified as chaotic, noisy, or quasi‐chaotic (Dennis et al. 2001, 2003; Ellner and Turchin 2005). This has ontological implications in terms of correctly understanding the process that generates the observed variability characteristic of the object under study. Even classical indicators for detecting chaos, such as the Lyapunov exponents, may be inappropriate in the presence of noise. To this end, a method for distinguishing noise from chaos has been proposed (Zunino et al. 2012), which is applied in the present study.

Attempts to investigate the role of periodic environmental forcings have been made by analyzing and generalizing the properties of simple dynamical models, which have shown that deterministic seasonal variability can generate cycles (Barraquand et al. 2017; Heilmann et al. 2016), repeated jumps between alternative attractors (Aron 1990; Keeling et al. 2001), and chaos (Benincà et al. 2015; Dakos et al. 2009; Heilmann et al. 2016; McCann and Yodzis 1994; Rinaldi et al. 1993; Sauve et al. 2020). Furthermore, noise can induce fascinating dynamics in ecosystems, such as stochastic resonance (Benzi et al. 1982; Gammaitoni et al. 1998; Occhipinti et al. 2024; Mantegna et al. 2000; Mantegna and Spagnolo 1994) and noise‐enhanced stability (Occhipinti et al. 2024; Mantegna and Spagnolo 1996; Spagnolo et al. 2004; Yu and Ma 2023; Zeng et al. 2015), which do not otherwise occur in deterministic dynamics. A recent review (Barraquand et al. 2017) summarized principal mechanisms currently recognized as playing major roles in explaining population cycling and aperiodic behavior and concluded, highlighting the need to expand the current research effort toward the exploration of higher dimension models, able to better capture the diversity of relationships and interdependency occurring within a food web (in contrast to the very simple models normally used so far), demography and trait evolution, and the role of environmental and demographic stochasticity.

In a very recent study, we addressed the first of those suggestions and explored about tens of thousands of different biogeochemical multinutrient multiplankton models of different complexity and configurations to conclude that biogeochemical models of realistic complexity rarely exhibit endogenous nonstationary (periodic or chaotic) dynamics (Occhipinti et al. 2023). In fact, in that study, in the absence of external forcing, most of the systems showed steady behavior or microfluctuations of very small (less than 5%) amplitude, possibly because of the presence of multiple negative feedbacks damping potential oscillations. This result suggests that aperiodic external forcings are a major ingredient, and possibly a necessary condition, to explain the fluctuations observed in nature.

Here we explore the point further by analyzing the response of three of those plankton models (expressing characteristic behaviors) under periodic and stochastic forcings of different intensity and colors (autocorrelation). In more detail, we investigate the impact on biogeochemical and plankton dynamics of periodic oscillations and of periodic oscillations plus noise in water temperature, which is one of the key factors controlling the seasonal variability of the plankton community (Rasconi et al. 2017; Wang et al. 2022). We use a reasonably complex state‐of‐the‐art multinutrient multiplankton functional type biogeochemical model (BFM; Vichi et al. 2020), which is currently used in the Copernicus Marine Service (CMS) for short‐term forecast and time model‐based reanalysis of biogeochemical properties (see Section 2.1). BFM simulates a planktonic food web of nine groups comprising phytoplankton, heterotrophic bacteria, microzooplankton, and mesozooplankton, accounts for biogeochemical cycling of carbon, phosphorus, nitrogen, and silicate, and describes the dynamics of the microbial loop and the evolution of dissolved and particulate organic matter.

The study of random temperature variations required the use of the stochastic version of the BFM (SBFM) presented and discussed in (Lazzari et al. 2021), which accounts for the effects of random variations by considering temperature as a stochastic process through an additive self‐correlated Gaussian noise. We modeled the random fluctuation of temperature over the seasonal cycle with an Ornstein‐Uhlenbeck process, which is characterized by a correlation time τ and noise intensity D. The correlation time τ represents the time scale over which the process retains memory of its previous states, with shorter τ indicating a faster “loss of memory” of the noise source. In other words, shorter the correlation time is, shorter the time interval after which the random fluctuations become again statistically independent. Conversely, a longer correlation time indicates a more persistent memory, resulting in smoother, more correlated fluctuations over time. We studied the model with five different correlation times, from the day scale up to the year scale, investigating the relationship between the dynamics of plankton and the noise correlation time.

Using a stochastic model, the temperature variations affect metabolic rates in all the trophic web in a more realistic way compared with the simple introduction of time dependence in the parameters as performed in previous works (Dakos et al. 2009; Heilmann et al. 2016; Rinaldi et al. 1993; Sauve et al. 2020). Marine systems are subject to random temperature fluctuations superimposed on the seasonal cycle. Therefore, to correctly and comprehensively model the dynamics of a marine ecosystem, one must account for such fluctuations, which can be modeled by a stochastic process (Lazzari et al. 2021). In particular, a stochastic process characterized by time‐correlated (red) noise is appropriate because marine environments have a memory for weather conditions (Barraquand et al. 2017; Steele and Henderson 1994; Lazzari et al. 2021). Furthermore, as a result of global warming, temperatures are increasing in both magnitude and degree of variability (Rasconi et al. 2017). Environmental fluctuations, especially when severe and sudden (e.g., marine heat waves), are expected to cause changes in plankton diversity and community structure, which are essential features for ecosystem functioning and trophic transfer (Graham and Vinebrooke 2009; Huang et al. 2021; Rasconi et al. 2017; Seuront et al. 1996). The relevance of strong weather events has also been documented for other marine organisms, for example, mussels (Wootton and Forester 2013; Benincà et al. 2015).

The present study is dedicated to understanding the causes of the aperiodic fluctuations observed in nature and is structured as follows. Section 2 describes the deterministic and stochastic biogeochemical models and presents the methods used to identify chaos (bifurcation diagrams (Sauve et al. 2020), Lyapunov exponents and the multiscale complexity–entropy causality plane (Zunino et al. 2012)). In Section 3, we describe the results of the effects of seasonality across various amplitudes of temperature oscillations and the influence of stochasticity. In Section 4, we discuss the results in light of the existing literature, and we summarize the novelty of this work.

## 2 Methods

### 2.1 The Deterministic Model

The Biogeochemical Flux Model (BFM) (Vichi et al. 2020), used here in a 0‐dimensional configuration (one point in the ocean), is formulated in terms of ordinary differential equations that account for the main biogeochemical processes in pelagic marine ecosystems (Sarmiento 2006). BFM is used for operational (Salon et al. 2019; Cossarini et al. 2021), process (Lazzari et al. 2012, 2016; Melaku Canu et al. 2015), and climate (Solidoro et al. 2022; Reale et al. 2022) studies at both the basin (Lazzari et al. 2012) and local (Lamon et al. 2014) scales, and to model the biogeochemical part, “Med‐BIO”, of “The Mediterranean Sea Monitoring and Forecasting Centre (Med‐MFC) system.” CMS regularly and systematically provides key reference information on the state, variability and dynamics of the ocean, marine ecosystems and sea ice for the global ocean and European regional seas. It has also been validated against various observational reference data (satellites, literature, climatology, BGC‐Argofloats) to quantify its consistency in simulating the main features of the biogeochemistry of the Mediterranean Sea and its accuracy in routinely reproducing observations at specific times and locations (Salon et al. 2019). Therefore, the BFM can be considered a state‐of‐the‐art model for marine biogeochemistry.

The BFM was developed to accurately reproduce the dynamics of the following plankton functional types (PFTs): primary producers (phytoplankton), predators (zooplankton), and decomposers (bacteria). Within these PFTs, more specific subgroups are identified to better describe the planktonic food web. Producers are divided into diatoms (P1), flagellates (P2), picophytoplankton (P3), and dinoflagellates (P4). Predators include nanoflagellates (Z6), microzooplankton (Z5), omnivorous mesozooplankton (Z4), and carnivorous mesozooplankton (Z3). Bacteria (B1) are responsible for the fundamental aspect of recycling organic compounds into inorganic constituents such as nitrates, phosphates, and silicates. In addition to describing the food web, the BFM also considers the major biogeochemical processes that characterize the dynamics of a pelagic marine ecosystem (e.g., cycles of nitrogen, phosphorus, silica, carbon, and oxygen in the water due to plankton activity). Solar irradiance (*I*) is considered constant to study the effects of the water temperature *T* variability.

In total, the BFM consists of a system of 54 nonlinear ordinary differential equations (ODEs), which describes 9 PFTs dynamics, and represent organic and inorganic substances fluxes within biogeochemical processes in the sea. For example, the evolution of the biomass of a generic primary producer (e.g., carbon in diatoms, $P1_{c}$) is described by:

(1) $$\frac{\mathrm{dP}1_{c}}{\mathrm{dt}}=f_{\mathrm{gpp}}\left(x_{\mathrm{BFM}},T,I\right)-f_{\mathrm{rsp}}\left(x_{\mathrm{BFM}},T,I\right)-f_{\mathrm{exc}}\left(x_{\mathrm{BFM}},T,I\right)-f_{\mathrm{prd}}\left(x_{\mathrm{BFM}},T\right)$$

The f‐s are continuous functions representing various biogeochemical fluxes associated with physiological processes. $x_{\mathrm{BFM}}$ is the 54‐dimensional state vector. Gross primary production gpp (expressed in mgCm^−3^d^−1^) is the entry point of carbon into the ecosystem and is essentially related to photosynthesis. Respiration (rsp) refers to the release of carbon (production of CO_2_) and combines basal and activity terms. Excretion processes (exc) are related to the metabolic activity of the cell and the balancing of the internal quota of carbon with respect to other elements. prd is predation or grazing by zooplankton. The f‐s are factorized into a number of regulatory functions

(2) $$f_{\mathrm{gpp}}\left(x_{\mathrm{BFM}},T,I\right)=r_{\max}f_{T}\left(T\right)f_{I}\left(I\right)f_{\mathrm{nut}}\left(x_{\mathrm{BFM}}\right)P1_{c}$$

where $r_{\max}$ is the maximum growth rate and it is phytoplankton specific, $f_{I}\left(I\right)$ is a light harvesting factor dropping to zero in the absence of light, and *f*_nut_ defines the limitation to growth caused by nutrient depleted conditions. The temperature regulation factor $f_{T}\left(T\right)$ is given by the following expression (Flynn 2001):

(3) $$f_{T}\left(T\right)=Q_{10}^{\frac{T-T_{\mathrm{ref}}}{T_{\mathrm{ref}}}}$$

where the dimensionless parameter $Q_{10}$ is 2.00 for all organisms except for the heterotrophic bacteria (B1), whose $Q_{10}$ is equal to 2.95 (Flynn 2001; Freund et al. 2006; Vichi and Masina 2009; Raven and Geider 1988). The reference temperature ($T_{\mathrm{ref}}$) is set to 10°C. The same temperature regulating factor ($f_{T}\left(T\right)$) acts on the other physiological processes. Consequently, higher/lower temperatures increase/decrease the overall metabolic rates and accelerate/decelerate the biogeochemical cycling of the system. Temperature dependence of phytoplankton growth rates can be much more complicated in reality, Lazzari et al. (2021), because, for instance, the functional relationship is usually nonmonotonic and has a species‐specific optimal temperature, but in the present case we are away from regimes of thermal limitation higher than 30°C. Although in modern metabolic theory the description of temperature regulation has moved from the $Q_{10}$ equation (Equation 3) to the Boltzmann–Arrhenius equation (Gillooly et al. 2001; Heine 2023), we have retained the classical formulation used in marine biogeochemical models (the $Q_{10}$ equation), since both methods have been shown to be equally suitable for correctly reproducing the plankton dynamics in the temperature range here considered (Sherman et al. 2016). The equations for zooplankton are similar to those for phytoplankton, with the gross primary production replaced by the grazing.

To mimic the seasonal variability of water temperature *T*, we assumed that the periodic (and deterministic) seasonal variations could be described by the following sinusoidal function:

(4) $$T\left(t\right)=T_{\det}\left(t\right)=\bar{T}-A_{y}\cos\left(\frac{2\mathrm{\pi t}}{\tau_{y}}\right)$$

where $\bar{T}$ = 15°C is the mean value of the temperature, $A_{y}$ is the amplitude of the annual cycle, t is the time, and $\tau_{y}=365\;\text{days}$ is the annual period. We studied the amplitude of oscillations in the interval [0, 15]°C with steps of 1°C.

For more details and the complete list of equations and processes included in the BFM, (Lazzari et al. 2012) and the BFM code manual (Vichi et al. 2020) are recommended.

### 2.2 The Stochastic Model

We used the stochastic BFM (SBFM) developed in (Lazzari et al. 2021), a modification of the deterministic BFM in which the temperature is considered as a stochastic process, consisting of a deterministic (periodic) component and a randomly fluctuating part. The noisy component, modeled as a virtual particle “moving” under the influence of a Gaussian self‐correlated noise, accounts for random environmental fluctuations, always present in a real ecosystem.

The following Langevin equation, characteristic of an Ornstein–Uhlenbeck process, describes the temperature evolution over time:

(5) $$T\left(t\right)=T_{\det}\left(t\right)+\chi_{T}\left(t\right)$$

(6) $$\frac{d\chi_{T}\left(t\right)}{\mathrm{dt}}=-\frac{\chi_{T}}{\tau }+\xi_{T}\left(t\right),\;\chi_{T}\left(0\right)=0$$

where $T_{\det}$ is the deterministic seasonally varying temperature described by Equation (4), $\chi_{T}$ is the noisy component of the temperature, τ the correlation time of the noise, and $\xi_{T}$ a white Gaussian noise with mean and covariance given, respectively, by

(7) $$E\left[\xi_{T}\left(t\right)\right]=0;\;E\left[\xi_{T}\left(t\right)\xi_{T}\left(t^{\prime }\right)\right]=\mathrm{D\delta}\left(t-t^{\prime }\right)$$

where D is the noise intensity expressed in °C/s and δ is the Dirac delta function. In the Ornstein–Uhlenbeck process (Equation 6), for sufficiently long times (t≫τ), the mean of $\chi_{T}$ tends to zero, while the variance tends to Dτ/2.

Therefore, the temperature in the SBFM is described by a superposition of seasonal periodic variations and autocorrelated noise (with correlation time τ). The choice of a colored noise stems from the fact that the time series of populations in real ecosystems are noisy and specifically “red”, that is, they are dominated by low frequencies (long‐term variations), or “white”, that is, they present no dominating frequency. In particular, terrestrial populations exist in a white noise environment (Steele and Henderson 1994), whereas marine populations inhabit a red noise environment (Blarer and Doebeli 1996; Steele and Henderson 1994; Lazzari et al. 2021). In SBFM, noise helps prevent extinction of some species, and the frequency of peaks in plankton populations is related to the noise timescale (Lazzari et al. 2021). Therefore, we examined the effects of stochastic temperature fluctuations with the following time scales $\tau =\left[10,\;30,90,180,365\right]\;\text{days}$, while D was chosen so that the long‐term variance remains constant *Dτ*/2 = 10^−2^°C. Due to the stochastic nature of the SBFM we only show average results obtained by averaging over 1000 realizations of the model, that is, solving numerically 1000 times the equations of the stochastic model (Lazzari et al. 2021).

### 2.3 Chaos Identification

We consider three approaches to classifying the temporal variability of the model. The first is based on the indicators used in a previous study on the same biogeochemical model (Occhipinti et al. 2023). The second approach is based on bifurcation diagrams and is used to analyze the response of the model based on a control parameter; in this case, the temperature oscillation amplitude. The first two approaches are only suitable for deterministic models; the complexity–entropy analysis is the third approach, which makes it possible to recognize the influence of chaos even in noisy systems. While this method can classify all kinds of variability (periodic, chaotic, noisy), it is computationally expensive, so it is used only for stochastic solutions, where it is strictly required.

In the first approach, the three indicators defined in (Occhipinti et al. 2023), that is, the coefficient of variation CV (indicator 1), the frequency spectrum (indicator 2), and the Lyapunov exponents λ's (indicator 3), are used to identify a solution as stationary, periodic, or chaotic. The coefficient of variation CV (indicator 1) is defined as

(8) $$\mathrm{CV}=\frac{\sigma }{\mu }$$

where σ is the standard deviation and μ is the mean of a plankton species biomass, both computed over the time t. CV is used to identify stationary and nonstationary solutions. An examination of the frequency spectrum (indicator 2) is used to identify periodic dynamics among the nonstationary solutions. The Lyapunov exponents λ (indicator 3) are used to identify periodic and chaotic solutions in the nonstationary subset, with a positive Lyapunov exponent being associated with chaos. Lyapunov exponents are computed with the algorithm of Wolf et al. (1985).

Because we adopted a numerical approach to perform this study, we had to use thresholds for the indicators: (i) a solution is considered stationary if $\mathrm{CV}<10^{-3}$ or it is identified as monotonic by indicator 2; (ii) a solution is considered periodic if $\mathrm{CV}>10^{-3}$ and $\lambda <10^{-3}$; (iii) otherwise the solution is chaotic. The threshold for CV is low enough to accurately detect nonstationary behavior (a lower threshold did not find any further nonstationary solutions).

Using the second approach, bifurcation diagrams, allows one to study the dynamic response of the system to the variation of $A_{y}$, the amplitude of the temperature oscillations. The diagrams are constructed in the following way (Pennisi et al. 2018): (i) for each value of $A_{y}$ the model is solved numerically; (ii) we construct the envelope of a plankton biomass time series with the constraint that two consecutive maxima and minima must be at least 30 days apart, using the last 10 years of the time series to neglect possible transient behavior; (iii) the maximum and minimum values are plotted as points corresponding to the $A_{y}$ of the respective model solution; (iv) this is repeated for each plankton species and each value of $A_{y}$. If a single point corresponds to a selected amplitude $A_{y}$, the biomass of the plankton species under investigation is at equilibrium. If there are two points, the biomass undergoes a cycle and assumes values between the two points of the diagram. An increasing number of points corresponding to a single $A_{y}$ is associated with an increasing number of cycles in the biomass, each with different maximum and minimum values. The number of points can increase up to fill a region of the diagram (a vertical segment); in this case, the local extrema take multiple values between the extremes, indicating chaotic behavior of the plankton species under study at the selected $A_{y}$. The choice of a 30 days separation between consecutive maxima/minima was made to ensure that the identified extrema correspond to meaningful, longer‐term dynamics rather than high‐frequency fluctuations or numerical noise. While intrinsic cycles or quasi‐cycles can indeed have periods shorter than 30 days, our focus here is on capturing the dominant seasonal and interannual variability in plankton biomass, which is more relevant to the ecological processes under study. This choice helps to filter out short‐term variability, that may obscure the underlying patterns of interest, and it is consistent with the timescales of the seasonal forcing and the ecological processes modeled. Sensitivity analyses (not shown) confirmed that the qualitative results of the bifurcation diagrams are robust to moderate variations on this timescale. Finally, we note that the short‐term variability is included in the noise signal, whose spectrum is continuous and contains in the present case, that is, self‐correlated noise, all frequencies from 0 up to the cutoff one. Since it is difficult to distinguish between chaos and a large number of cycles with different maximum and minimum, we use the bifurcation diagram for a qualitative study of the response of the planktonic ecosystem to the increase in the amplitude of the temperature oscillations, and we associate a quantitative difference between periodic and chaotic behavior through the computation of the Lyapunov exponents. Therefore, we use colors to distinguish, in the bifurcation diagram, the points that refer to a solution for which at least one species presents $\lambda >10^{-3}$ (in blue) from those which do not fulfill this condition (in orange).

**FIGURE 1:**
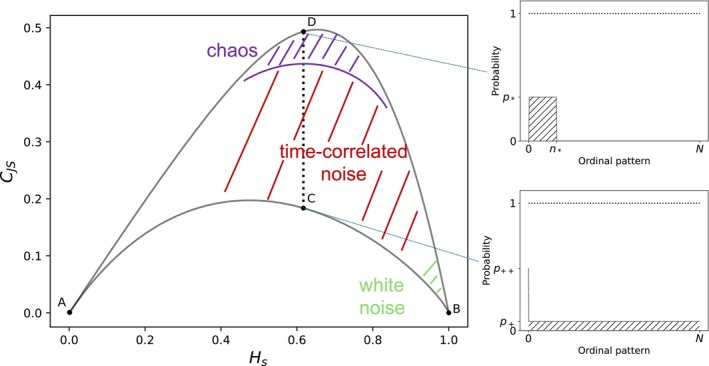
Example of multiscale complexity–entropy causality plane (left panel): Any probability function is mapped in a point within the two curves. Dirac delta‐shaped probability distributions are mapped in the point A. Uniform distributions, which indicate white noise dominance, are mapped in the point B. Other permitted points represent intermediate conditions. In particular, points with same information entropy $H_{s}$ could have different complexity values $C_{\mathrm{JS}}$. Point C represent a probability distribution with the lower complexity for the given $H_{s}$ and corresponds to a uniform distribution ($p=p_{+}$) with the exception of a single ordinal pattern with higher probability ($p=p_{++}$). Point D represents a distribution with same $H_{s}$ as point C but with the highest $C_{\mathrm{JS}}$, this distribution is uniform ($p=p_{*}$) on a subspace of the possible ordinal patterns from 1 to $n_{*}$ and zero elsewhere. Approximate position of chaotic systems, and colored and white noises are depicted in different colors.

Lyapunov exponents, as calculated using the algorithm of Wolf et al. (1985), are unable to detect chaos when noise is present. A visual method to distinguish chaos from stochasticity has been developed by studying two information theory quantifiers, the normalized Shannon entropy $H_{S}$ and the statistical complexity measure $C_{\mathrm{JS}}$ (Rosso et al. 2007; Zunino et al. 2012). They characterize the diversity and the correlational structure of a time series, respectively. The normalized Shannon entropy is defined as (Zunino et al. 2012)

(9) $$H_{S}\left(p\right)=\frac{S\left(p\right)}{S_{\max}}$$

where $S\left(p\right)=-\sum_{i=1}^{M}p_{i}\ln p_{i}$ is the Shannon logarithmic information measure (in ecology Shannon diversity) for a discrete variable x which can take a finite number M of possible values with corresponding probabilities $p_{i}\in p=\left(p_{1},\ldots ,p_{M}\right)$, $S_{\max}=S\left(p_{e}\right)=\ln M$ is a normalization constant and $p_{e}=\left(1/M,\ldots ,1/M\right)$ is the uniform distribution. The fact that the normalized Shannon entropy is maximized by the uniform distribution means that a time series has a higher entropy if its values are in a random order. To compute the probabilities p from the time series, we use the permutation method introduced by Bandt and Pompe (Bandt and Pompe 2002). In this approach, the time series is divided into overlapping segments of length d, called embedding dimension, separated by a time delay ϵ, called embedding delay. d refers to the number of values forming the segment. The embedding delay ϵ is the time separation between the values extracted from the time series and physically corresponds to a multiple of the sampling time of the signal to be analyzed. Consequently, different time scales are taken into account by changing the embedding delay (Zunino et al. 2012). Each segment is ranked to form an ordinal pattern, that is, the values are sorted in ascending order, and the corresponding permutation is recorded. For example, a segment $\left(x_{t},x_{t+\epsilon },x_{t+2\epsilon }\right)=\left(4.2,3.1,5.6\right)$ would be mapped to the permutation pattern $\left(2,1,3\right)$ since the second value is the smallest, followed by the first, and then the third. The process is repeated for all segments in the time series, and the relative frequency of each unique permutation is calculated. These frequencies directly form the probability distribution p associated with the time series dynamics, with each entry representing the likelihood of observing a specific ordinal pattern. The choice of embedding parameters, d and ϵ, influences the resulting distribution p. A common choice is $d\in \left[3,7\right]$ and ϵ=1 (Bandt and Pompe 2002), but these can be adjusted depending on the system's dynamics. The embedding dimension controls the number of possible ordinal patterns (d!), while the delay parameter ϵ defines the temporal separation between elements in the pattern. These parameters allow for capturing system dynamics across different time scales (Zanin and Olivares 2021). We choose d=6, following (Zanin and Olivares 2021). This method is robust to noise and invariant under monotonic transformations of the data, making it particularly useful for real‐world applications (Zunino et al. 2012). Further details can be found in the Data S1. The statistical complexity measure is defined as (López‐Ruiz et al. 1995)

(10) $$C_{\mathrm{JS}}\left(p\right)=Q_{J}\left(p,p_{e}\right)H_{S}\left(p\right)$$

where $Q_{J}\left(p,p_{e}\right)=Q_{0}J\left(p,p_{e}\right)$ is called disequilibrium, $J\left(p,p_{e}\right)=S\left(\left(p+p_{e}\right)/2\right)-S\left(p\right)/2-S\left(p_{e}\right)/2$ is the Jensen–Shannon divergence, and $Q_{0}=1/\max J\left(p,p_{e}\right)$ is a normalization constant. $Q_{J}$ is minimized by the uniform distribution, while $H_{S}$ is maximized, therefore a time series has a high value of statistical complexity if its values are halfway between random and order. Two distribution vectors p can be associated with the same value of $H_{S}$ but with different values of $C_{\mathrm{JS}}$, therefore for a given $H_{S}$ value the $C_{\mathrm{JS}}$ can range from $C_{\mathrm{JS}}^{\min}$ to $C_{\mathrm{JS}}^{\max}$ (Rosso et al. 2007). Evaluating $C_{\mathrm{JS}}$ offers additional insights into the correlative characteristics of a probability distribution that entropy alone does not provide (see Figure 1). In fact, the closer the time series approach $C_{\mathrm{JS}}^{\max}$ the higher distance from the uniform distribution, indicating correlative features and therefore maximal theoretical complexity for the given entropy $H_{S}\left(p\right)$. The normalized Shannon entropy and the statistical complexity measure are computed using the Python package *ordpy* (Pessa and Ribeiro 2021), which uses the permutation method of Bandt and Pompe to estimate a probability distribution p from a time series (Bandt and Pompe 2002; Zunino et al. 2012). Using this methodology, these quantifiers are usually referred to as permutation entropy $H_{S}$ and permutation statistical complexity $C_{\mathrm{JS}}$ (Zunino et al. 2012). We follow this notation. Both quantifiers are a function of the embedding delay ϵ and the embedding dimension d.

The dynamical behavior of a time series can be estimated by examining the multiscale complexity–entropy causality plane (Zunino et al. 2012), which is the parametric curve described by the permutation statistical complexity (vertical axis) and the permutation entropy (horizontal axis) estimated from a time series with the embedding delay as a parameter, such that the curve follows the changes in the embedding delay. This approach provides a versatile tool for discriminating dynamics at different time scales (Zunino et al. 2012). Chaos is associated with intermediate $H_{S}$ values, while $C_{\mathrm{JS}}$ reaches larger values. It has been proposed to identify chaos with $H_{S}$ between 0.45 and 0.7 and $C_{\mathrm{JS}}$ near the maximum (Zanin and Olivares 2021), and we adopt this assumption. Stochastic processes corresponding to delta‐correlated (white) noise signals are associated with $H_{S}$ and $C_{\mathrm{JS}}$ close to 1 and 0, respectively. Stochastic processes corresponding to time‐correlated (colored) noise signals are associated with intermediate permutation entropy and intermediate statistical complexity values (Rosso et al. 2007). A schematic representation of the complexity–entropy causality plane and the regions occupied by chaos, white noise and time‐correlated noise is shown in Figure 1. The presence of a maximum for the permutation statistical complexity at an embedding delay together with an increasing behavior of the permutation entropy around this scale is a necessary (but not sufficient) condition for the presence of chaos (Zunino et al. 2012). We eliminated the underling periodic signal from the time series before computing the quantifiers: The study of a pure chaotic, noisy, or noisy plus chaotic signal in the complexity–entropy plane, is simpler when there is no superimposed cyclic behavior.

It is interesting to investigate whether the composition of the food web described by the BFM changes. When the biomass of a species drops below $10^{-4}\mathrm{mgC}\;m^{-3}$, it is no longer experimentally observable. Therefore, we define the solutions in which at least one species with biomass below this value occurs as extinct, which is a misuse of notation since, even if the species is not observable, this does not imply that it is extinct.

We performed 20‐year simulations of the BFM (and SBFM) with 5000 timesteps per year equivalent to a timestep of 6307.2 s.

## 3 Results

### 3.1 Deterministic Seasonal Forcing

In the unforced BFM, it is possible to observe nonstationary (periodic or chaotic) dynamics. The initial concentration of phosphate (${\mathrm{PO}}_{4}$), the assimilation efficiency $\eta_{z}$ and the excreted fraction of uptake $\beta_{z}$ of microzooplankton are found to be determinant in the occurrence of nonstationary solutions (Occhipinti et al. 2023), hence they can play the role of bifurcation parameters. We performed a preliminary study over the stability of the unforced model ($A_{y}$ = 0°C in Equation 4) varying ${\mathrm{PO}}_{4}$ in the interval $\left[0.01,0.20\right]\;\text{mmol}\;P\;m^{-3}$ and $\beta_{z}$ in $\left[0.35,0.65\right]$, the intervals are chosen to include all the possible kind of solutions (extinct, stationary, periodic, chaotic) defined in the Methods section.

**FIGURE 2:**
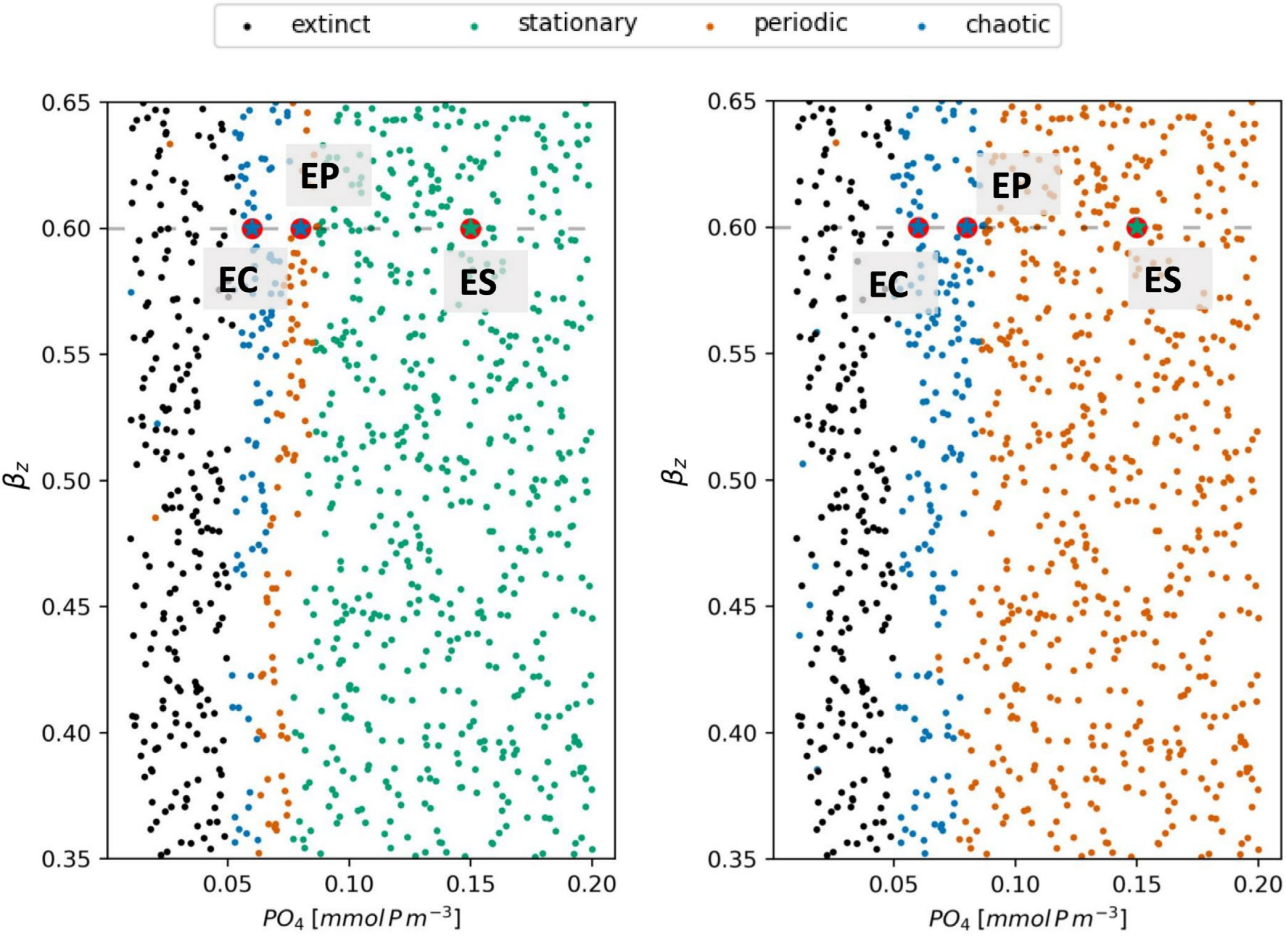
Approach 1. Sensitivity analysis for the stability of the BFM as response to the variation of the initial concentration of phosphate ${\mathrm{PO}}_{4}$ and the microzooplankton excreted fraction of uptake $\beta_{z}$. 1000 simulations are carried out, each with a random value of the parameters (${\mathrm{PO}}_{4}$, $\beta_{z}$). Colors are used to show the dynamics of the solutions, which are identified as described in Section 2.3. *Left panel*: No seasonal temperature oscillations, *Right panel*: Seasonal temperature oscillations with amplitude $A_{y}=5^{o}C$. The stars inscribed in red circles indicate three chosen configurations: ES with stationary (0.15, 0.60), EP with periodic (0.08, 0.60) and EC with chaotic (0.06, 0.60) solutions of the unforced model. “Extinct” indicates a condition such that the biomass of at least one species drops below $10^{-4}{\text{mgCm}}^{-3}$, becoming no longer experimentally observable.

We performed 1000 simulations to explore randomly the parameter space $\left({\mathrm{PO}}_{4},\beta_{z}\right)$, the dynamical behavior of each solution is shown in Figure 2. Therefore three configurations (shown in Figure S1a) are selected by such analysis in order to study the effect of seasonal forcing over stability regimes when the unforced model would present stationary (${\mathrm{PO}}_{4}=0.15,\beta_{z}=0.60$), periodic (${\mathrm{PO}}_{4}=0.08,\beta_{z}=0.60$), or chaotic (${\mathrm{PO}}_{4}=0.06,\beta_{z}=0.60$) regimes (these selected configurations are labeled with stars in Figure 2). For clarity we assign an acronym to each configuration: (i) The configuration resulting in a solution of the deterministic BFM with no periodic forcing, which expresses stationary dynamics will be called configuration ES, which stands for endogenous stationary dynamics and is associated to the parameter values (${\mathrm{PO}}_{4}=0.15,\beta_{z}=0.60$); (ii) the configuration resulting in a solution of the deterministic BFM with no periodic forcing, which expresses periodic dynamics will be called configuration EP, which stands for endogenous periodic dynamics and is associated to the parameter values (${\mathrm{PO}}_{4}=0.08,\beta_{z}=0.60$); (iii) the configuration resulting in a solution of the deterministic BFM with no periodic forcing, which expresses chaotic dynamics will be called configuration EC, which stands for endogenous chaotic dynamics and is associated to the parameter values (${\mathrm{PO}}_{4}=0.06,\beta_{z}=0.60$).

A similar sensitivity analysis (see Figure 2) is performed for the seasonally forced model with an amplitude $A_{y}$ = 5°C characteristic of the Mediterranean Sea (Vichi and Masina 2009). The periodic solutions clearly predominate in the forced model. At this value of the amplitude of the temperature oscillations, the forcing is capable of pushing the configuration EP into chaos and, in general, of pushing most configurations expressing periodic endogenous dynamics into chaos in the forced model.

**FIGURE 3:**
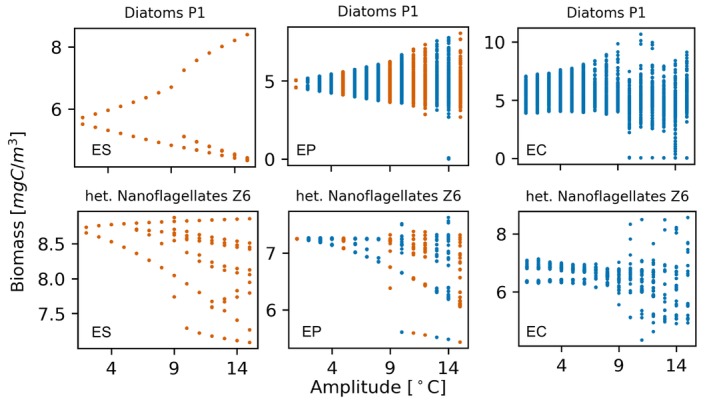
Approach 2. Bifurcation diagrams for one phytoplankton species (Diatoms P1) and one zooplankton species (heterotrophic nanoflaggelates Z6) in the configuration ES (left panels), EP (mid panels), and EC (right panels). Similar results are obtained for the other modeled plankton species. Orange dots correspond to periodic dynamics ($\lambda <10^{-3}$) and blue dots to chaotic dynamics ($\lambda >10^{-3}$). In the configuration ES chaos never occurs.

To understand the relationship between chaos and the amplitude of temperature oscillations, each of the three configurations (ES, EP, EC) is studied under the forcing of the seasonal oscillations of temperature, varying the amplitude in the interval [0, 15]°C. The amplitude $A_{y}$ is used to produce a bifurcation diagram in order to observe the dynamical behavior of ES, EP, and EC as a function of the seasonality (diagrams shown in Figure 3). In the configuration ES, the increase in the amplitude of temperature oscillations is associated with an increase in the number of cycles of the plankton biomass, that is, at different times of the year the biomass peaks have different magnitudes, indicating that in the phase space the system periodically visits different attractors associated with different biomass concentrations. Therefore, the solutions of a model with stationary endogenous dynamics and subjected to a periodic forcing can be multiperiodic but are never observed to be chaotic (see Figure 3 left panels). In the configuration EP, the seasonal forcing is capable of throwing the ecosystem into chaos for most amplitudes (see Figure 3 mid panels). The Lyapunov exponents show that, for certain values of the amplitude, the chaotic behavior is lost and the ecosystem adopts multiperiodic dynamics. The seasonal temperature forcing cannot stabilize the configuration EC to (multi‐)periodic dynamics, so that chaos occurs for any value of the amplitude (see Figure 3 right panels).

**FIGURE 4:**
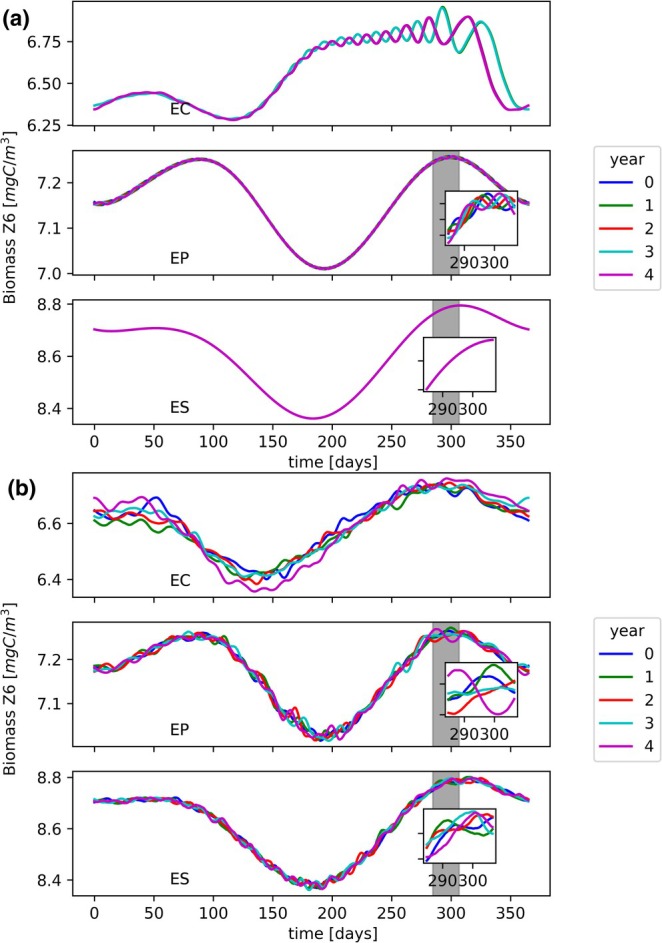
Biomass time series for the heterotrophic nanoflagellates (the other species presented similar behaviors), shown for the last 5 years of a 20‐year simulation. Each year is overlaid to show the annual variations. In bottom panels, the stationary configuration ES, in mid panels the periodic configuration EP and in top panels the chaotic configuration EC. (a) Deterministic model: Seasonal temperature oscillations with amplitude $A_{y}=5^{o}C$, (b) Stochastic model: Seasonal temperature oscillations with amplitude $A_{y}=5^{o}C$ and noise time scale τ=30days. The gray regions are zoomed in the subplot boxes.

We have further investigated whether chaos is related to year‐to‐year variability in population density. We found that, when chaos occurs, there is annual variability (of a few days or more) of density peaks in plankton biomass (see Figure 4a), while for the configuration ES, the cycles induced by the temperature oscillations are locked to their annual frequency.

### 3.2 Stochastic Forcing

**FIGURE 5:**
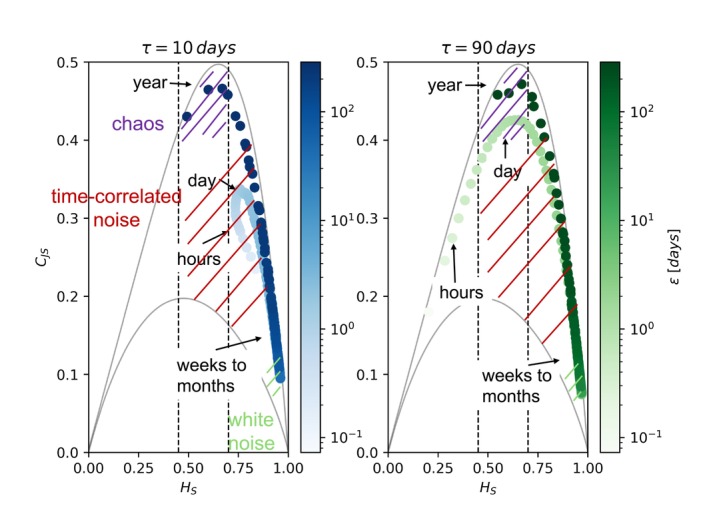
Approach 3. Multiscale complexity–entropy causality plane for solutions of the stochastic model in the configuration ES with different values of the noise correlation time τ. The biomass of diatoms (P1) is analyzed. The gray lines represent the theoretical maximum and minimum $C_{\mathrm{JS}}$. The chaotic solutions reach the maximum $C_{\mathrm{JS}}$ in the interval $H_{S}\in \left[0.45,0.70\right]$, which indicates chaos. Each point in the planes is characterized by a value of the embedding delay ϵ, which is indicated by the color and the broader time scale of ϵ by the text boxes. For τ=10days chaos is identified only at the year scale, while for τ=90days both at the day and year scales.

We repeated the analysis of the configuration ES, EP, EC using the SBFM. In the stochastic configuration we graphically investigated the occurrence of chaos by the use of the multiscale complexity–entropy causality plane, shown for the configuration ES in Figure 5 (for the configurations EP and EC. see Figures S4 and S5). Permutational entropy and statistical complexity metrics show that random temperature fluctuations can induce chaotic dynamics, even in solutions with stationary endogenous behavior. This suggests that stochastic forcing has the potential to generate chaotic behavior independently of the endogenous deterministic dynamics of the model. In the three configurations, chaos was identified for any correlation time τ of the stochastic forcing, supporting the results for a wide range of time‐correlated noise.

Since the results for noise correlation time τ=30,90,180,365days are similar, in Figure 5 we reported the multiscale complexity–entropy causality plane of the solutions with τ=10days and τ=90days in the ES configuration (for τ=30,180,365days see Figure S3). Similar results are obtained with the configuration EP (see Figure S4). In the configuration EC, the range of embedding delays ε (time scales of the time series) in which chaos is identified increases compared with the configurations ES and EP (see Figure S5). This suggests that endogenous chaos is more difficult to be masked by noise than chaos induced by external forcings.

The time scales (ϵ) at which the chaotic structures develops are in the range of 1–2 days with the exception of τ=10days, and around the annual time scale for all the cases. In fact with τ=10days, the curve at lower ϵ has permutation entropy above 0.8. This high entropy value suggests that, at shorter embedding delays, the noise with a small correlation time τ dominates the dynamics, significantly modifying the deterministic structure. However, for larger embedding delays, the fast fluctuations are averaged out, allowing the detection of chaotic features. For noise with a larger correlation time τ, the fluctuations are smoother and the deterministic behavior can be detected even on time scales of the order of days.

The stochastic forcing is able to produce an annual variability in the plankton biomasses for each of the three configurations studied (see Figure 4b). This occurs for any of the noise correlation times considered (not shown). Such a behavior could be expected since the amplitude of the stochastic forcing (noise intensity) undergoes an annual variability itself.

**FIGURE 6:**
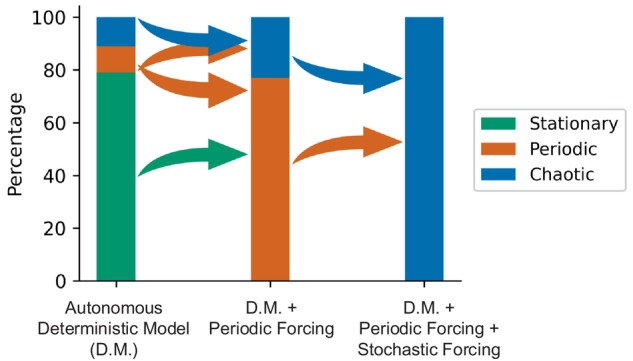
Bar chart summarizing the occurrence of stationary (green), periodic (orange), and chaotic (blue) dynamics in marine biogeochemical models. In the autonomous deterministic model (D.M.), external forcings are not present and the endogenous dynamics are mostly stationary. If an environmental periodic forcing is added to the model (D.M. + Periodic Forcing), the stationary endogenous dynamics are transformed into periodic dynamics and sometimes the periodic dynamics are transformed into chaos. If the environmental noise over the periodic forcing is also taken into account (D.M + Periodic Forcing + Stochastic Forcing), chaos is always identified, at least for some time scales. The ratio between the different types of dynamics is calculated using the sensitivity analysis in Figure 2 for the first two columns and using the multiscale complexity–entropy planes for configurations ES, EP, and EC at 5 time scales of noise for the third column.

The study shows that in a deterministic model, the endogenous dynamics are usually stationary and rarely exhibit periodic or chaotic behavior. However, the introduction of periodic forcing can transform stationary dynamics into periodic ones and periodic dynamics into chaos. Moreover, the addition of noise together with periodic forcing leads to chaos in the model, regardless of the original endogenous dynamics. These results are summarized in Figure 6.

## 4 Discussion and Conclusions

Numerous studies on the consequences of seasonal forcing on plankton communities found a relationship between seasonality and chaotic dynamics (Benincà et al. 2015; Dakos et al. 2009; Heilmann et al. 2016; Rinaldi et al. 1993; Sauve et al. 2020). Several simplifications, however, were made; for example, trophic webs with few species were considered, and model parameters were simply modulated with time dependency to parameterize temperature seasonal variability and its impact on metabolic rates. While these simplified models are sufficient to show the route to chaos, they may overlook ecological complexities that influence real‐world dynamics. Furthermore, it is important to analyze the role of chaos and noise in models used in operational oceanography to provide ecosystem forecasts. To address this, we chose a state‐of‐the‐art biogeochemical model (the BFM), which explicitly represents the effects of temperature on plankton through regulatory functions (see Equations 2 and 3). This complexity is necessary to assess whether the chaotic behavior identified in simpler models arises under more realistic ecological conditions. By exploring 15 different values of temperature oscillation amplitude, 5 noise correlation times, and three model configurations (ES, EP, EC) associated with stationary, periodic, and chaotic endogenous dynamics, we can investigate the interaction between the autonomous model and exogenous forcing in a system that captures essential biological and chemical processes. With this approach, we can determine whether realistic mechanisms can drive an ecosystem into chaos.

Predator–prey models (Rinaldi et al. 1993) show that seasonal forcings, even with small amplitudes, can increase the number of attractors of a system and lead to the coexistence of several (quasi‐)periodic solutions, as clearly observed in the bifurcation diagrams (see Figure 3). Seasonal forcing is able to lead periodic endogenous dynamics into chaos (Heilmann et al. 2016; Rinaldi et al. 1993). Since this phenomenon occurs in simple up to high‐dimensional models, like that presented here, it is possible to hypothesize that periodical forcings can induce chaos as a general mechanism present in nature. Our results suggest that a marine biogeochemical model of realistic complexity predicts that plankton do not behave chaotically when subjected to a seasonal external forcing of arbitrary strength, in a realistic range of values, if the endogenous dynamic is stationary (see Figure 3 left panels), as found in physiologically structured population plankton models (Heilmann et al. 2016) but contrary to what was found in previous studies based on simplified models (Rinaldi et al. 1993). The absence of chaos may be linked to the long oscillation period employed. Plankton, with a reproductive cycle lasting days, may not perceive temperature variations over seasons. Previous studies have established a correlation between plankton behavior and external forcing timescales (e.g., Benincà et al. 2011; Lazzari et al. 2021). We performed 200 additional simulations using model configuration ES, randomly varying temperature oscillation periods (1–365 days) and amplitudes (1°C–10°C). None exhibited chaotic dynamics, indicating periodic forcing cannot throw stationary endogenous dynamics into chaos. Together with temperature, solar irradiance is a key factor driving the temporal and spatial variability of plankton dynamics (Sathyendranath et al. 1989). In addition to investigating the effects of temperature oscillations, we conducted a preliminary study on the impact of solar irradiance oscillations. Our results revealed a scenario similar to the one illustrated in Figure 2. This similarity suggests that it may not be the oscillation of water temperature responsible for periodic or chaotic dynamics in planktonic populations, but rather the broader oscillations in the environmental conditions that plankton experience.

The BFM has been shown to rarely exhibit endogenous oscillations with amplitudes large enough to be experimentally observable (CV > 10^−2^), but up to one‐third of the realistic parameter configurations of the model results in nonstationary solutions with a CV of magnitude order $10^{-3}$ (Occhipinti et al. 2023). Therefore, we found that chaos induced by seasonal forcing takes place only in about 30% of the parameter configurations: The ones characterized by endogenous periodic or chaotic oscillations. This percentage is lower than that observed in the records examined in a recent meta‐analysis (80% of the time series considered showed chaos; Rogers et al. 2022). Although the level of organization at which chaos is studied may have an influence on its observation, (Rogers et al. 2023) has found chaos less common for taxonomic aggregates (such as the functional types of our model) than for individual species. We wish to highlight that it is not possible to directly relate frequency in parameter space to frequency in field observations, as the parameter distribution in nature is unknown and a different model could lead to different results. The relevance of this analysis relies on the fact that it provides support for one mechanism that could trigger chaos in plankton communities. In this context, random fluctuations turn out to be an additional ingredient that contributes to trigger chaos.

Noise has been shown to cause irregularities in cycles (Barraquand et al. 2017; Burgers 1999). We found that time‐correlated noise applied to water temperature is able to push the ecosystem toward chaos, even if the endogenous dynamics are stationary (see Figure 5). Therefore, the combination of stochastic and periodic environmental forcings can trigger chaos in planktonic communities, regardless of the underlying dynamics of the model. The fact that the marine environment is intrinsically stochastic (Steele and Henderson 1994) leads therefore to the conclusion that the environmental random fluctuations can contribute to inducing chaos in a wide range of scenarios.

The Lyapunov exponents of time series are related to its degree of predictability, with longer predictability being associated with a smaller exponent. It turns out that there is no monotonic relationship between the degree of predictability and the oscillation amplitude of temperature (see Figure 3). An increase in amplitude is not always associated with an increase in the Lyapunov exponent. Strong temperature oscillations (e.g., $A_{y}$ = 15°C in the EP configuration) can result in periodic dynamics of the time behavior of the plankton community. The fact that the amplitude of the forcing alone is not sufficient to explain the transition to chaos is consistent with previous studies (e.g., Dakos et al. 2009; Sauve et al. 2020).

A seasonal forcing tends to lock the frequency of population density oscillations at the same frequency of the seasonal cycle or integer multiples of it (Scheffer et al. 1997), as shown by the annual oscillations (see Figure S1b). However, annual variability in species concentrations is observed in nature (e.g., in plankton blooms, insect communities, soil fauna, and annual plants). This variability could be explained by the interaction among species in a seasonally forced environment, even if the forcing does not exhibit annual variations (Dakos et al. 2009). We observe that interannual variability appears when nonstationary (periodic or chaotic) endogenous dynamics are combined with external forcing. This is especially true for the values of the temperature oscillation amplitude for which the ecosystem is chaotic. Therefore, the observed annual variability seems to be related to chaotic behavior of the ecosystem. Clearly, environmental conditions inducing interannual variability in temperature, or other external regulating factors, could also drive the variability of the ecosystem, as shown by the study on the effects of random temperature fluctuations (see Figure 4b).

The study carried out on the permutation entropy $H_{S}$ and the permutation statistical complexity $C_{\mathrm{JS}}$ suggests that chaos is correctly detected only at certain embedding delays, when the system is exposed to a noise source. Therefore, a data limitation in the observations may bias the characterization of the dynamics and confuse chaos with noise. This result is consistent with the hypothesis that the rarity of chaos in past observations is due to methodological bias rather than biological reasons (Rogers et al. 2022). Chaos was initially introduced in ecology to elucidate population fluctuations through deterministic nonlinear interactions. However, given that all ecosystems are inherently subjected to noise, debate persists regarding the applicability of defining a system as chaotic in the presence of stochastic fluctuations (Dennis et al. 2001, 2003; Ellner and Turchin 2005). Our research reveals that deterministic chaos can indeed emerge as the leading behavior of the dynamics of a stochastic system, at least at certain time scales. Although the fluctuations observed in nature cannot be strictly deterministically explained, it is crucial to acknowledge that they are not strictly stochastic either. Consequently, we argue that chaos theory remains a relevant framework for ecological studies. Moreover, noise not only obscures deterministic signals, but can also help to reveal underlying deterministic patterns; this perspective finds support in previous studies (King et al. 2004; Desharnais et al. 2023).

Our analysis supports the hypothesis that chaos can occur in plankton communities and highlights possible mechanisms able to lead to chaos in real marine ecosystems. The first mechanism, consisting of the interplay between periodic endogenous dynamics and periodic external forcing, requires an existing particular endogenous dynamic. The second mechanism, consisting of the interplay between stochastic external forcings and periodic external forcings, does not require any particular endogenous dynamics, paving the way to broader applicability. Recognizing and understanding these dynamics is crucial for advancing our comprehension of ecological systems, enabling accurate predictions, and fostering sustainable practices in the face of a globally changing environment.

## Author Contributions

**Guido Occhipinti:** conceptualization (lead), data curation (lead), formal analysis (lead), investigation (lead), methodology (lead), software (lead), validation (equal), visualization (lead), writing – original draft (lead). **Cosimo Solidoro:** conceptualization (equal), funding acquisition (equal), validation (equal), writing – review and editing (equal). **Roberto Grimaudo:** writing – review and editing (equal). **Davide Valenti:** conceptualization (equal), writing – review and editing (equal). **Paolo Lazzari:** conceptualization (equal), funding acquisition (equal), methodology (supporting), supervision (equal), validation (equal), writing – review and editing (equal).

## Conflicts of Interest

The authors declare no conflicts of interest.

## Supporting information


**Data S1:** ece371930‐sup‐0001‐Supinfo.pdf.

## Data Availability

The code for the BFM and its manual can be freely downloaded at bfm‐community.eu. The configurations of the model and the python code used in their analysis can be found at https://doi.org/10.5281/zenodo.12699670 (Occhipinti 2024).
