# Supplementary material for: Plankton Communities Behave Chaotically Under Seasonal or Stochastic Temperature Forcings
Source: Ecol Evol. 2025 Aug 16;15(8):e71930. doi: 10.1002/ece3.71930 (PMC12357154; doi:10.1002/ece3.71930)
Supplement: Supplementary file 1 — Data S1: ece371930‐sup‐0001‐Supinfo.pdf. [file ECE3-15-e71930-s001.pdf]

Supporting Information for

Plankton communities behave chaotically under seasonal or stochastic temperature forcings

Guido Occhipinti, Cosimo Solidoro, Roberto Grimaudo, Davide Valenti, Paolo Lazzari

Guido Occhipinti
E-mail: gocchipinti@ogs.it

This PDF file includes:

Supporting text
Figs. S1 to S5
SI References

Supporting Information Text

Model configurations. In this work, three configurations of the model parameters are selected from a sensitivity analysis study (see Fig. 2 in the main manuscript). The first configuration, referred to as the ES configuration, has parameters $PO_4 = 0.15$ and $\beta_z = 0.60$, presenting stationary endogenous dynamics in the deterministic unforced model. The second configuration, referred to as the EP configuration, has parameters $PO_4 = 0.08$ and $\beta_z = 0.60$, presenting periodic endogenous dynamics in the deterministic unforced model. The third configuration, referred to as the EC configuration, has parameters $PO_4 = 0.06$ and $\beta_z = 0.60$, presenting chaotic endogenous dynamics in the deterministic unforced model. Here, we report the time series of the nine plankton species for the unforced model (Fig.S1a) and the model with periodic forcing with amplitude $A_y = 5°C$ (Fig.S1b) for the three configurations ES, EP, and EC.

Bifurcation diagrams. Here, we provide a figurative representation (Fig.S2) of the construction of bifurcation diagrams, as described in the Methods section of the main manuscript. We analyze the biomass time series of diatoms in the EP configuration of the deterministic model.

The effectiveness of our method is demonstrated through the construction of the bifurcation diagram for a well-known dynamical system, the logistic map [1]. Mathematically, it is expressed as

$$x_{t+1} = a\, x_t(1 - x_t)\,, \tag{1}$$

where x_t is a number between zero and one, representing the ratio of the existing population to the maximum possible population, and a is the reproduction rate. For $(a > 3)$, the solutions are periodic, while chaos sets in at $(a \approx 3.56995)$.

Permutation entropy and permutation statistical complexity. The permutation entropy H_S (Eq. (9) in the manuscript) and permutation statistical complexity C_{JS} (Eq. (10) in the manuscript) are computed from probability distributions. These probabilities are estimated from a time series using the permutation method of Bandt and Pompe [2]. How the probabilities p_i are constructed from a time series and for a given embedding delay ϵ and embedding dimension d is illustrated by the following example [2]. The embedding delay ϵ is the time separation between the symbols (i.e. the values extracted from the time series), d refers to the number of symbols forming the ordinal pattern. We take a time series with 7 elements $\boldsymbol{\psi} = (\psi_0, ..., \psi_t, ...\psi_7) = (4, 7, 9, 10, 6, 11, 3)$ and embedding delay $\epsilon = 1$, so we consider all elements of the time series. We use $d = 2$ as the embedding dimension, which means that we need to extract pairs of neighbors from $\boldsymbol{\psi}$ and confront them in the following way. We can identify $M = 6$ pairs in $\boldsymbol{\psi}$, 4 of which are characterized by $\psi_t < \psi_{t+1}$, represented by the permutation 01. The remaining 2 pairs are characterized by $\psi_t > \psi_{t+1}$ and represented by the permutation 10. Therefore, the set of probabilities constructed with $d = 2$ is $\mathbf{p} = (4/6, 2/6)$. Similarly, with $d = 3$ we can identify $M = 5$ triads, $(4, 7, 9), (7, 9, 10), (9, 10, 6), (10, 6, 11), (6, 11, 3)$. The first two triads are characterized by $\psi_t < \psi_{t+1} < \psi_{t+2}$ and are identified by the permutation 012, $(9, 10, 6)$ and $(6, 11, 3)$ are identified by the permutation 201, $[10, 6, 11]$ by the permutation 102. The set of probabilities constructed with $d = 3$ is $\mathbf{p} = (2/5, 2/5, 1/5)$.

The multiscale complexity-entropy-causality plane constructed from the time series of diatom biomass from the SBFM in configuration ES for $\tau = 30, 180, 365\,s$ is shown in Fig.S3. Chaos is identified at the day and at the year sampling scales (ϵ) for any noise correlation time τ. The plane for configuration EP is shown in Fig.S4. Similarly, the plane for configuration EC is shown in Fig.S5. When the noise is characterized by $\tau = 10d$, chaos can not be identified at time scales (ϵ) in the days range for any possible endogenous dynamics (ES, EP and EC), while it is identified at the year scale for any τ. The largest interval of time scales at which chaos can be identified occurs when the endogenous dynamics are chaotic (configuration EC).

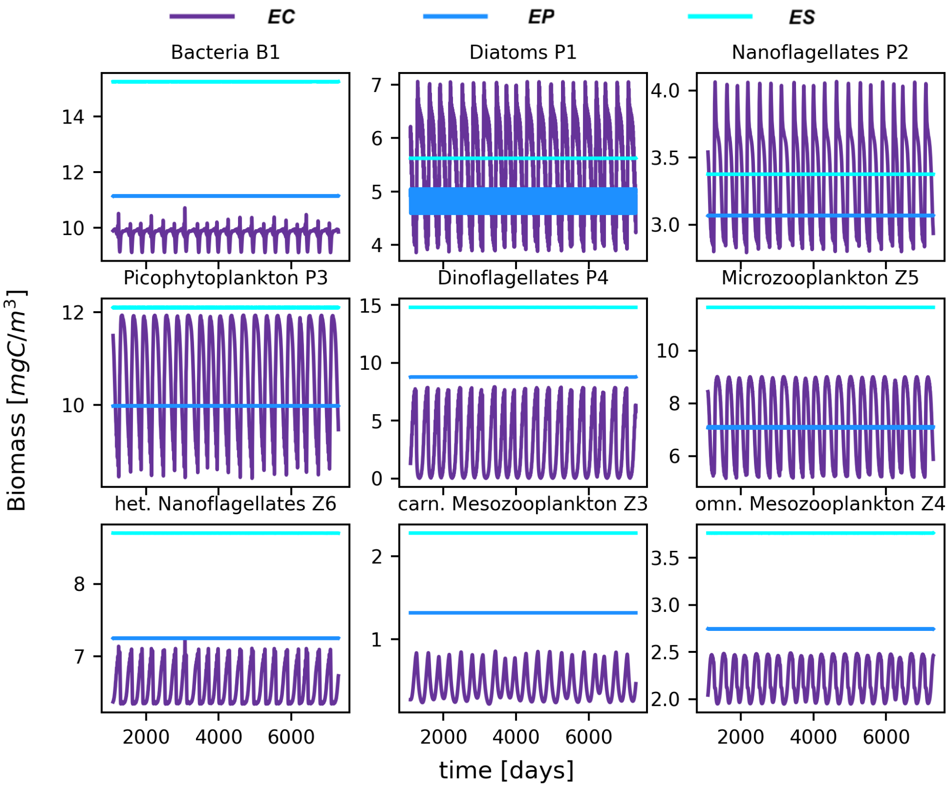

(a)

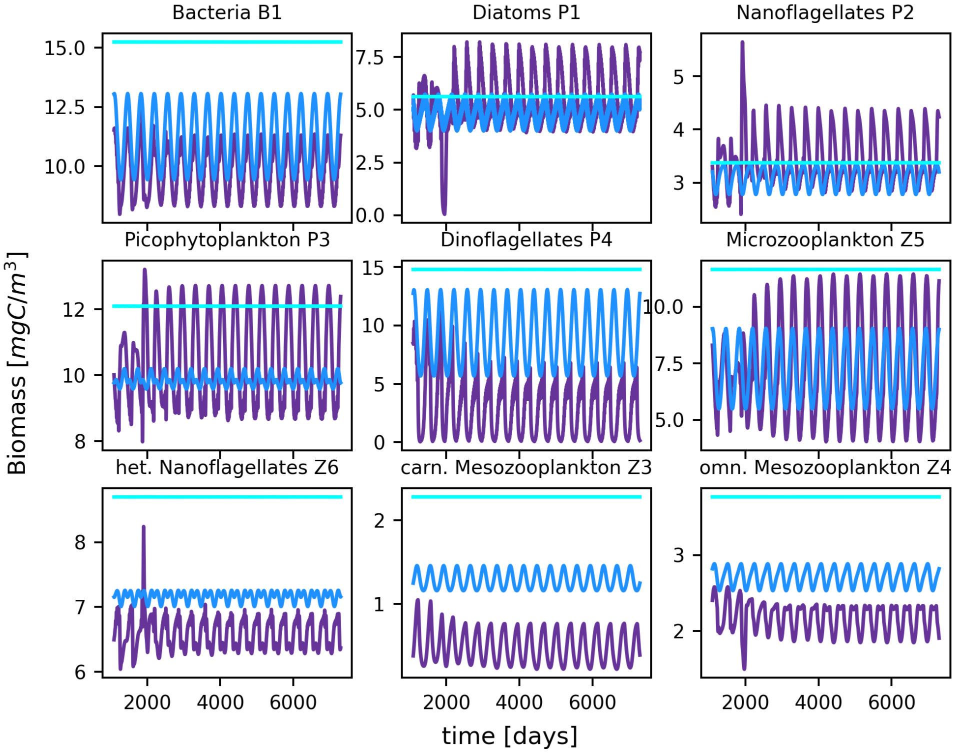

(b)

Fig. S1. Model configurations. Biomass time series for the BFM biological species, corresponding to the last 17 years of the simulation to avoid the transient period. In cyan the stationary configuration ES, in blue the periodic configuration EP, in purple the chaotic configuration EC. (a) No seasonal temperature oscillations, (b) seasonal temperature oscillations with amplitude $A_y = 5°C$.

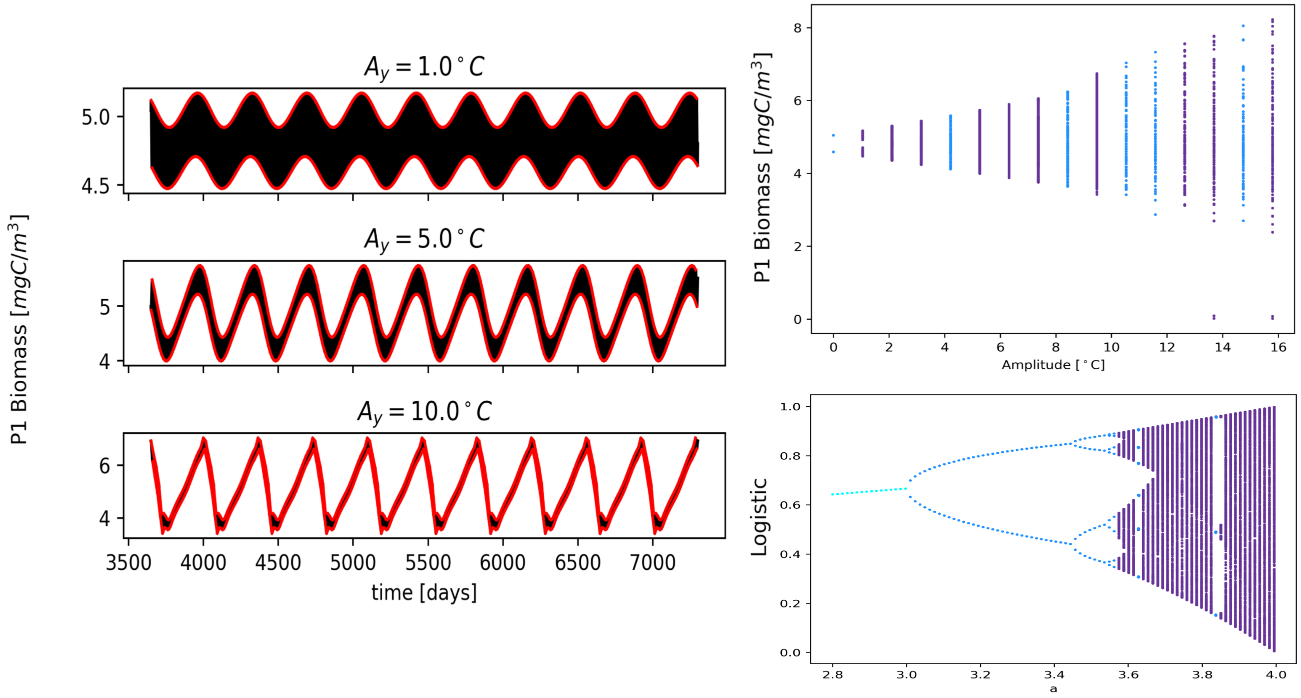

Fig. S2. Bifurcation diagrams. Scheme of the procedure used to construct a bifurcation diagram from time series. *Left panel:* Envelope of diatom biomass shown in red for 3 different amplitudes of temperature oscillations. The biomass time series is in black. *Top right panel:* Bifurcation diagram constructed from the maxima and minima defined by the envelope curve. The blue dots correspond to periodic dynamics, the purple dots to chaos, and are identified using Lyapunov exponents. *Bottom right panel:* Bifurcation diagram of the logistic map. The light blue dots correspond to stationary dynamics, the blue dots to periodic dynamics, the purple dots to chaos.

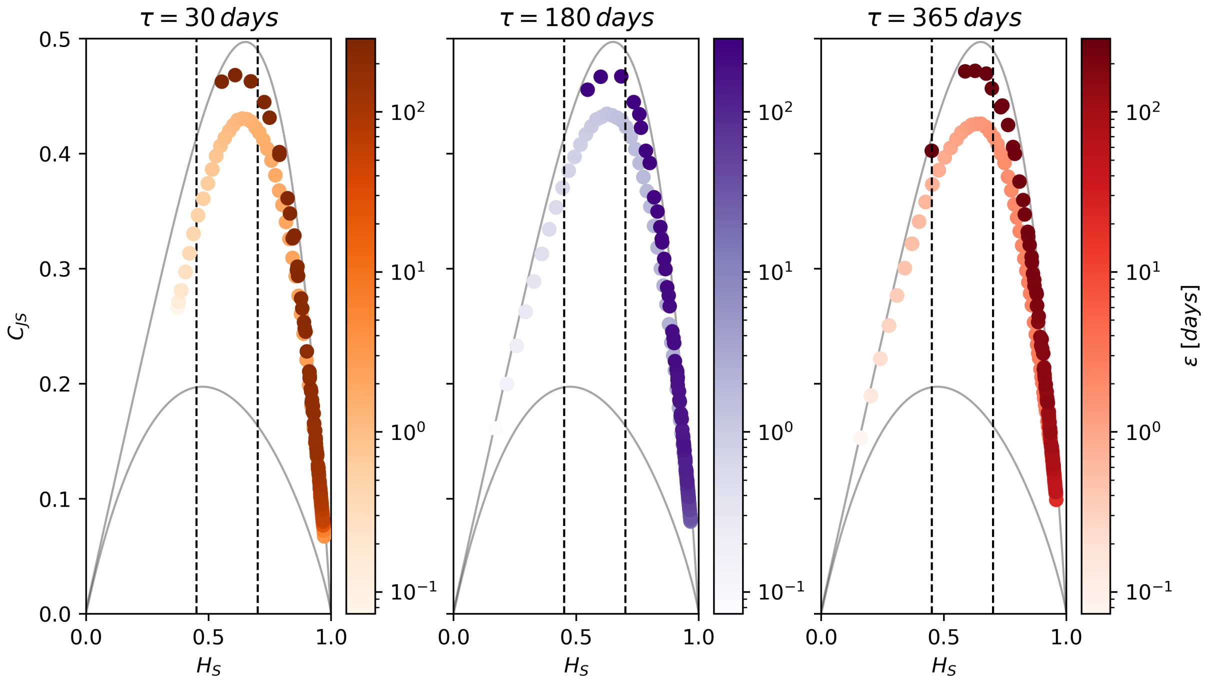

Fig. S3. Multiscale complexity-entropy causality plane for solutions of the stochastic model in the configuration ES with different values of the noise correlation time τ. The biomass of diatoms (P1) is analyzed. The grey lines represent the theoretical maximum and minimum C_{JS}. The chaotic solutions reach the maximum C_{JS} in the interval $H_S \in [0.45, 0.70]$, which indicates chaos. Each point in the planes is characterized by a value of the embedding delay ϵ, which is indicated by the color. Chaos is identified both at the day and year scales.

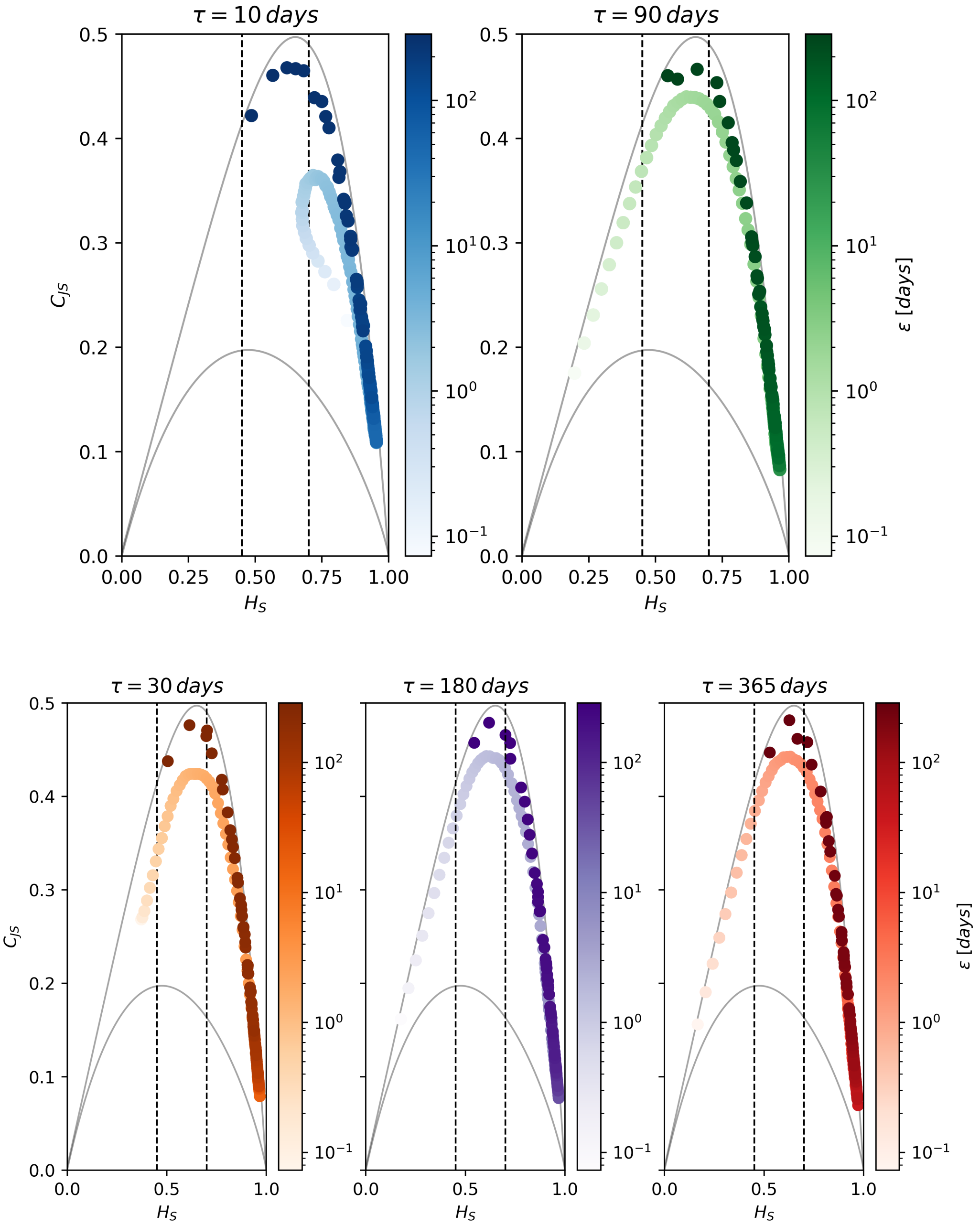

Fig. S4. Multiscale complexity-entropy causality plane for solutions of the stochastic model in the configuration EP with different values of the noise correlation time τ. The biomass of diatoms (P1) is analyzed. The grey lines represent the theoretical maximum and minimum C_{JS}. The chaotic solutions reach the maximum C_{JS} in the interval $H_S \in [0.45, 0.70]$, which indicates chaos. Each point in the planes is characterized by a value of the embedding delay ϵ, which is indicated by the color. Chaos is identified both at the daily and annual scales for each τ, except for $\tau = 10d$, when chaos is only recognised on the annual scale.

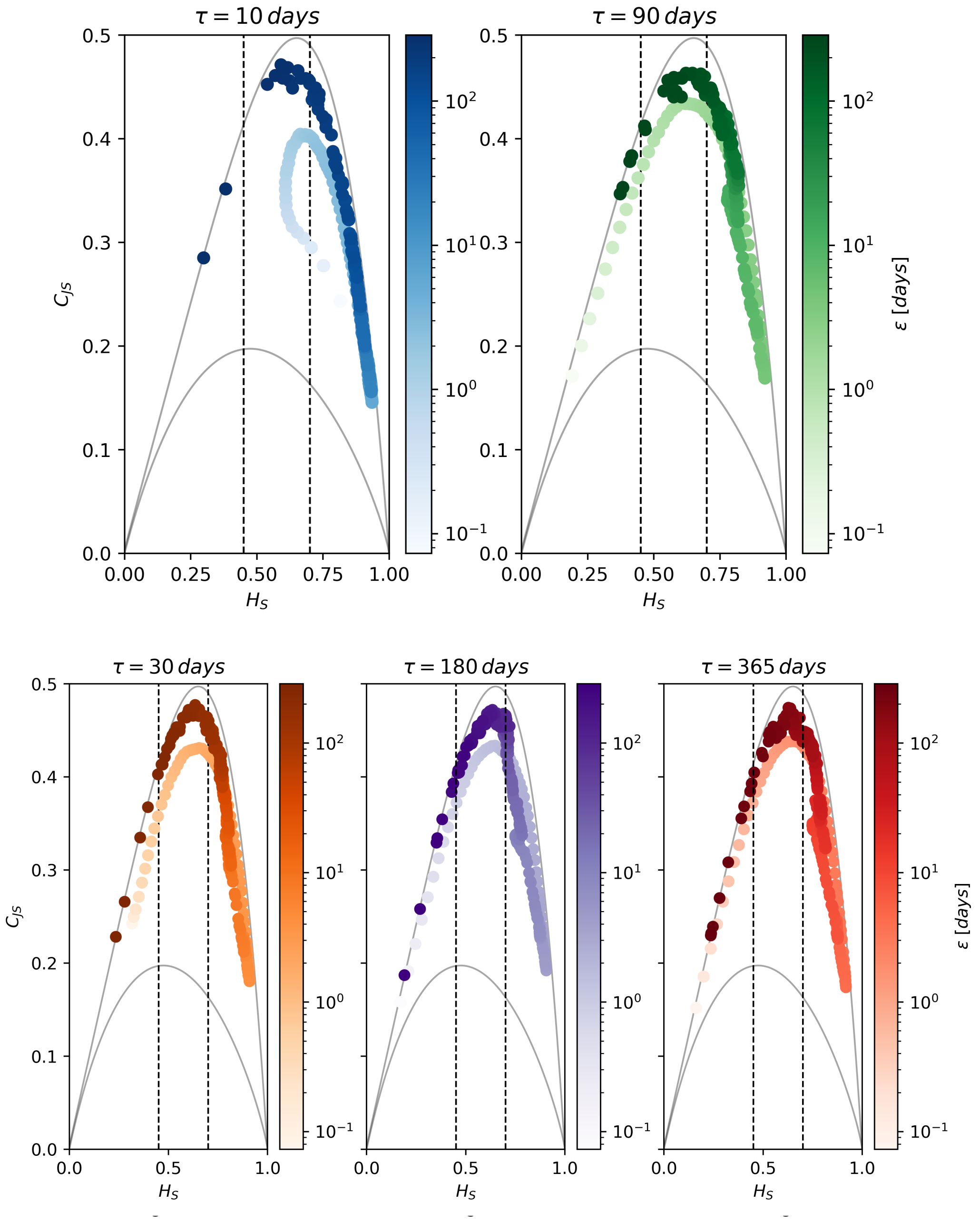

Fig. S5. Multiscale complexity-entropy causality plane for solutions of the stochastic model in the configuration EC with different values of the noise correlation time τ. The biomass of diatoms (P1) is analyzed. The grey lines represent the theoretical maximum and minimum C_{JS}. The chaotic solutions reach the maximum C_{JS} in the interval $H_S \in [0.45, 0.70]$, which indicates chaos. Each point in the planes is characterized by a value of the embedding delay ϵ, which is indicated by the color. Chaos is identified both at the daily and annual scales for each τ, except for $\tau = 10d$, when chaos is only recognised on the annual scale.

References

1. Robert M. May. Simple mathematical models with very complicated dynamics. *Nature*, 261(5560):459–467, June 1976. ISSN 0028-0836, 1476-4687. .
2. Christoph Bandt and Bernd Pompe. Permutation entropy: A natural complexity measure for time series. *Physical Review Letters*, 88(17):174102, Apr 2002. ISSN 0031-9007, 1079-7114. .
